# The politest form of racism: sexual and reproductive health and rights paradigm in Canada

**DOI:** 10.1186/s12978-021-01117-8

**Published:** 2021-03-09

**Authors:** Ieman M. El-Mowafi, Abdiasis Yalahow, Dina Idriss-Wheeler, Sanni Yaya

**Affiliations:** 1 Interdisciplinary School of Health Sciences, University of Ottawa, Ottawa, Canada; 2 School of International Development and Global Studies, Faculty of Social Sciences, University of Ottawa, 120 University Private, Ottawa, ON K1N 6N5 Canada; 3 The George Institute for Global Health, Imperial College London, London, UK

**Keywords:** Sexual and Reproductive Health and Rights, Reproductive Justice, Racism, Canada

## Abstract

The Canadian national identity is often understood as what it is not; American. Inundation with American history, news, and culture around race and racism imbues Canadians with a false impression of egalitarianism, resulting in a lack of critical national reflection. While this is true in instances, the cruel reality of inequity, injustice and racism is rampant within the Canadian sexual and reproductive health and rights realm. Indeed, the inequitable health outcomes for Black, Indigenous and people of color (BIPOC) are rooted in policy, research, health promotion and patient care. Built by colonial settlers, many of the systems currently in place have yet to embark on the necessary process of addressing the colonial, racist, and ableist structures perpetuating inequities in health outcomes. The mere fact that Canada sees itself as better than America in terms of race relations is an excuse to overlook its decades of racial and cultural discrimination against Indigenous and Black people. While this commentary may not be ground-breaking for BIPOC communities who have remained vocal about these issues at a grassroots level for decades, there exists a gap in the Canadian literature in exploring these difficult and often underlying dynamics of racism. In this commentary series, the authors aim to promote strategies addressing systemic racism and incorporating a reproductive justice framework in an attempt to reduce health inequities among Indigenous, Black and racialized communities in Canada.

## Background

In this commentary series, we challenge the existing sexual and reproductive health and rights (SRHR) paradigm in research, health promotion, policy, and patient care in the Canadian context, and stress the importance of shifting towards one which incorporates reproductive justice and considers the impacts of structural racism [1]. We begin with the authors’ key assumptions underlying how SRHR work transpires in Canada, elucidate the importance of incorporating a reproductive justice framework [2], and outline key questions on the impact of anti-Black and anti-Indigenous racism on four domains of SRHR.

These commentaries are founded on the following assumptions, well grounded in the literature: (i) racism, not race, is the determinant of health, and racism is the reason why that distinction is seldomly made [3]; (ii) the lack of disaggregated data by race in Canada is prohibitive for Indigenous, Black and racialized communities to conduct and implement evidence-based interventions to improve and promote health equity [4]; (iii) current research, programming and decision making are not predominantly being conducted or led by Indigenous, Black and/or racialized researchers or community members; rather, SRHR research and interventions studies are mainly led by white women within academic, philanthropic or government agencies [5]; and (iv) these structural issues are the result of, and maintained by current assumptions of race and health that are rooted in white supremacy [2, 6–8]. As a result, the current paradigm cannot effectively address the needs of Indigenous, Black and racialized populations and we have reached a point where the situation has become unbearable. Stakeholders working in this space need to be transparent in their own assumptions that “white is normal”, or that racial equity in health already exists; the current methods and programming—rooted in white supremacy—do not incorporate a reproductive justice framework that meets the needs of marginalized populations [9, 10].

The Reproductive justice framework is an intersectional framework that includes social justice and human rights [1, 11]. It was founded in the 1990s, by a grass-roots organization led by womxn of color (WoC) in the United States. [11, 12]. In order to better understand the undoubtable interlinks between structural racism and sexual and reproductive health (SRH) outcomes among Indigenous, Black and racialized communities, these intersectional frameworks warrant more attention among researchers, governments, funders and advocates working in this sphere [2, 8, 12].

Through a multi-sectoral lens, we begin this series of commentaries by questioning key assumptions of benevolence, altruism and trust in the way research is conducted, health promotion is carried out, health services are delivered, and how the health sector is regulated. Each of these domains and the problems therein are complex and require a “deep dive” to be adequately understood. As such, we introduce the concepts in this commentary as a springboard for detailed discussions in subsequent commentaries.

## Research: knowledge is power

In the domain of research, we have identified three central issues that continue to sustain systems of oppression within academic and research settings which perpetuate gaps in SRHR research in Canada. The research field favours the privileged class [13]; indeed, white women have historically been the beneficiaries of these positions of power, including health research [14]. This privilege is self-perpetuating and prevents us from fixing the ills in society; it also contributes to the construction and fortification of barriers to Indigenous, Black, and racialized students from entering post-secondary or post-graduate education [13, 14].

The second issue we have identified surrounds funding governance and its fatal effect on equitable programming [15]. As a component of health research, funding matrices exist within the systems and structures of oppression that continue to subjugate Indigenous, Black and racialized voices [5]. Canadian Institutes of Health Research (CIHR) has indicated that they are willing to engage in an important conversation about issues of equity, diversity and inclusion in the research funding system [16, 17]. Funding agencies and academic entities must reflect on application and adjudication processes and adjust grant objectives and criteria to be inclusive of the lived experiences and responsibilities disproportionately affecting racialized people.

The third issue surrounds knowledge mobilization and translation. Knowledge mobilization operates by determining who can know, how we know, and what counts as knowledge [18]. This impacts the research questions asked, the data analyzed, and the frameworks or theories used for analysis; all of which affect the knowledge produced and used [18]. We argue that knowledge mobilization has been grounded in historical injustices. While there is a need for disaggregated race-based data, the history of manipulating, misrepresenting, and oppressing BIPOC communities under the guise of “research” requires that race-based data collection is ethically and culturally sound and led by members of its community [19]. Knowledge translation practices like academic publishing continue to disenfranchise BIPOC and other marginalized groups, leading to further inequities in research, funding, and inclusion in decision making spaces [20, 21].

## Policy: law as the determinant of health

In the domain of policy, we have identified three main areas of concern: (i) historically,  laws in Canada have been inherently applied through anti-Indigenous and anti-Black racism [22]; (ii) the application of law in Canada is founded on anti-Indigenous and anti-Black racism [22], and; (iii) the avenues for reformation lay within the legal domain [23]. Canadian law and policy have historically upheld the subjugation of racialized communities, contributing to the inequitable allocation of health-protective resources across class and race [23–25].

At a distal level, racialized communities have been effectively criminalized due to inequitable application of the law, resulting in disproportionate representation in incarcerated populations [26]. Additionally, the legal system is rife with bureaucracy, legalese, and expenses that make it unnecessarily difficult to navigate [27]. As a result, racialized communities experience stress and anxiety during interactions with the criminal justice and legal systems [23, 27]. In order to mitigate these effects, legal reforms and policy changes need to consider the health disparities that have arisen from racist historical contexts. In addition, federal and provincial legislatures need to collaborate, rather than obfuscate, to identify which specific issues can be addressed in their respective or collective jurisdictions.

The law is the most direct tool to repair historical injustices and address health disparities, with a focus on SRH outcomes for Black, Indigenous and racialized communities [23]. The combined jurisdiction of federal and provincial legislatures allows a range of possible reforms including hospital governance and evidence-based oversight to close the gaps in health outcomes for marginalized communities in Canada. We urge Canadian legislatures to enshrine distributive determinants of health within the law to further Indigenous, Black and racialized communities’ rights to health and reproductive autonomy. Additionally, we call upon the judiciary to challenge racist, ableist and white supremacist structures, policies and regulations that perpetuate health inequities among Indigenous, Black and racialized communities.

## Health promotion: who’s reproductive health matters?

In the domain of health promotion (HP), we have identified three central issues arising from historical injustices that require fundamental restructuring, if not radical dismantling. First, the theoretical and structural frameworks from which HP operates in Canada are centered around white, middle class, university educated folks who are mostly removed from the health issues they aim to address [28, 29]. Indeed, these dynamics are damaging and support a status quo which harms those—namely, Indigenous, Black and racialized communities—who continue to be neglected by institutions, decision makers, and policymakers [30, 31]. While it is important to acknowledge the positionality and power imbalances that operate in HP spaces, it is necessary for the field as a whole to weigh the consequences–intended or unintended–on SRHR outcomes at a population level.

The second issue surrounds discussions of whose knowledge and skills are celebrated, promoted, and most importantly, funded. Beneficiaries of the status quo are heralded with accolades, even as their ‘gold standard’ bodies of work are founded on the theft, appropriation, or subjugation of the work of marginalized others. While many others have discussed traditional healing practices, we must recognize the knowledge and skills not currently being used in health promotion practices, deemed instead as ‘alternative’, ‘cultural’ or ‘spiritual’[23]. Indeed, in 2015, Indigenous leaders addressed in the reconciliation report, the importance of incorporating traditional healing practices to address the effects of colonization on health outcomes among Indigenous communities [32].

Third, population health interventions are inequitably applied such that they diverge into one of two paths: health promotion or criminalization. While health promotion aims to improve the lives of its population, we argue that these systems often target, criminalize and oppress specific demographics, while embarking on harm reduction efforts for majority white communities. While some of the policies and practices may have not explicitly been designed to lead to inequity and injustice, it is nevertheless the duty of population health researchers to bring these shortcomings to the public discourse, offer critiques, and present just solutions for community healing.

## Patient care- first, do no harm

The current health care system in Canada is riddled with inequity, barriers to entry, and a lack of representation, stemming from historically inequitable policies in medical and health education, accreditation and governing bodies, and in hospitals and health centers [33, 34]. The current reality of patient care for BIPOC patients is one of mistrust, neglect and selective empathy. Indeed, the history of health professionals has been rooted in slavery for Black womxn, colonialism for Indigenous womxn and continued racism for both.

The first issue is the inequities within education and health institutions, including colleges and boards, and hospitals and health centers, and how these inequities contribute to overall poorer health outcomes for BIPOC populations. In 2016, Hoffman and colleagues documented the racial bias in pain management among white medical students and residents confirming the commonality of neglect of BIPOC clients [35]. Furthermore, the current system of self-regulation of colleges continues to disenfranchise those calling for systemic change, thereby favoring the status quo.

The second area of concern is the systems sustaining the barriers experienced by BIPOC and other marginalized populations to have the support, opportunity, and privilege necessary for admission to health education and accredited institutions. The current system favors the privileged, and rarely makes space for a diverse range of health care providers [36]. The violent history of the medical profession towards BIPOC communities have made positive provider–client relationships challenging to develop, highlighting the need for equitable pathways of entry for marginalized populations into health service professions [37].

Lastly, issues surrounding medical bias have been explored and documented across Canada; yet incidences of medical racism continue to be a regular occurrence for BIPOC patients, especially in SRHR spaces. A recent incident of fatal medical racism occurred in September 2020, with the death of an Indigenous woman in a Québec hospital [38]. Until there are safeguards in place for BIPOC patients in hospitals, the current system is ill-equipped to maintain the safety and wellbeing of all patients. The recent pandemic has further exposed systemic racism in health provision and access—ethnic and racial minorities in Canada continue to experience disproportionate health outcomes which predate the current COVID-19 crisis [39]. Racism—and its intersections with class and gender inequality—has made BIPOC “more exposed and less protected” to harms during the pandemic (40).

## Conclusion

In the following series of articles, the authors will discuss the importance of health researchers, decision-makers, and providers to frame their work within their specific historical and political contexts and consider how colonization, structural racism, and intersectional oppression molds the systems that interact with and impact racialized communities. The standard of “white as normal” in health programming for Indigenous, Black and racialized populations in Canada is a colonialist hangover that continues to perpetuate disparities in health outcomes within our communities. The current approach to SRHR governance in Canada is characterized by prejudice in design and capriciousness in delivery, along with unilateral white control. As a result, the SRHR field as it stands does not meet the needs of Indigenous, Black and racialized individuals in this country. This is evidenced by the way in which research is conducted, policies are designed, care is delivered, and health promotion is applied. There is a scarcity in the mechanisms to foster legal accountability, and a silent ground of powerful, complacent, and fearful individuals to address discrimination.

## Data Availability

Not applicable.

## Abbreviations

BIPOC: Black, Indigenous and People of Colour
SRH: Sexual and reproductive health
SRHR: Sexual and reproductive health and rights
WoC: Womxn of color
